# Application of an integrated multi-criteria decision making AHP-TOPSIS methodology for ETL software selection

**DOI:** 10.1186/s40064-016-1888-z

**Published:** 2016-03-02

**Authors:** Mohamed Hanine, Omar Boutkhoum, Abdessadek Tikniouine, Tarik Agouti

**Affiliations:** Departement of Computer Science, Laboratory of Engineering and Information Systems, Faculty of Sciences Semlalia, Cadi Ayyad University, Marrakesh, Morocco; Team of Telecommunications and Computer Networks, Faculty of Sciences Semlalia, Cadi Ayyad University, Marrakesh, Morocco

**Keywords:** ETL, ETL software selection problem, Business Intelligence, MCDM (multi-criteria decision making), AHP (analytic hierarchy process), TOPSIS (technique for order preference by similarity to ideal solution)

## Abstract

Actually, a set of ETL software (Extract, Transform and Load) is available to constitute a major investment market. Each ETL uses its own techniques for extracting, transforming and loading data into data warehouse, which makes the task of evaluating ETL software very difficult. However, choosing the right software of ETL is critical to the success or failure of any Business Intelligence project. As there are many impacting factors in the selection of ETL software, the same process is considered as a complex multi-criteria decision making (MCDM) problem. In this study, an application of decision-making methodology that employs the two well-known MCDM techniques, namely Analytic Hierarchy Process (AHP) and Technique for Order Preference by Similarity to Ideal Solution (TOPSIS) methods is designed. In this respect, the aim of using AHP is to analyze the structure of the ETL software selection problem and obtain weights of the selected criteria. Then, TOPSIS technique is used to calculate the alternatives’ ratings. An example is given to illustrate the proposed methodology. Finally, a software prototype for demonstrating both methods is implemented.

## Background

Business Intelligence (BI) is a field of information systems architecture. It allows implementing the means to collect, transform and restore data to assist decision-makers in enterprises. The heart of BI is based on data warehouses powered by ETL, which is generally a part of BI and is usually the starting point for each project. With the development of the use of BI, ETL becomes a critical factor affecting the success of a BI project. In the surveys conducted to determine important questions touching decision-makers and designers of BI projects (Simitsis et al. 2009; Wyatt et al. 2009), ETL software has been ranked among the highest priorities (Kimball and Caserta 2004).

One of the important and initial activities of a BI project is the selection of the most appropriate ETL software. In other words, the chosen software must be in accordance with the organizational goals and has to maximize the benefits of the organization. Then, choosing ETL software is a wise choice that limits the costs and risks associated with projects.

Moreover, decision making in the field of Business Intelligence software selection such as ETL, has become more complex due to a large number of software products in the market. For choosing the most appropriate software, in this regard, ISO/IEC 9126-1 (2001) has established six quality characteristics for any software products: Functionality, Reliability, Usability, Efficiency, and Maintainability.

Due to the multi-criteria nature for selecting ETL software, multi-criteria decision making (MCDM) has been found to be a powerful and suitable technique to solve this type of selection problems. The analytical model integrated with AHP (Analytical Hierarchy Process) method and TOPSIS (Technique for Order Preference by Similarity to Ideal Solution) will help to determine the right judgment in ETL software selection based on decision-makers’ specific requirements. This paper proposes an integrated AHP-TOPSIS model considering both qualitative and quantitative factors. In this respect, AHP can be very useful in involving several decision-makers with multiple conflicting criteria to arrive at a consensus in the decision making process. On the other side, TOPSIS technique is used to calculate the alternatives ratings.

The remainder of the paper is organized as follows. The second section gives a brief overview of existing methods and studies for software selection. In section three, the methods used in the proposed decision-making methodology are presented. The proposed methodology is concisely explained in the fourth section. As regards the next section, for better understanding of the methodology, an empirical study is illustrated, and sensitivity analysis is highlighted. The implementation of a software prototype for demonstrating the proposed methodology is given in section six. Finally, conclusions and further research are offered in the last section.

## Software selection review

The problem of the choice of BI tools is one of the strategic decisions that have a significant impact on business performance. With the evolution of decision support systems, the making decision becomes increasingly critical. However, ETL software selection is considered to be a highly important research issue in BI (Simitsis et al. 2009), but it has not yet received much attention in research as further as research on this subject is necessary. One of the main motivations of this research is the absence of evidence in the literature that such papers do not employ a methodological approach-such as AHP-TOPSIS-for evaluating and selecting ETL software.

The state of the art is very rich by various methods suggested for the selection problem (Amiri 2010). All the methods can be classified in four different categories: MCDM (multi-criteria decision making) is the first category which contains different methods such as: AHP, ANP (Analytical Network Process), PROMETHEE (preference ranking organisation method for enrichment evaluations), ELECTRE (ELimination and Choice Expressing Reality) TOPSIS etc. Wei et al. (2005) used the AHP method to identify priority in selecting ERP System. Similarly, Yigit et al. (2014) developed an interactive model using AHP to facilitate the selection of Web-based learning object software. Besides, Göztepe (2012) applied ANP method to appraise and select the best Operating system with regard to organizational factors and strategic performance metrics. Behzadian et al. (2012) asserted a TOPSIS based model for multi criteria decision making in another study.

The second category concerns mathematical programming methods. Data Envelopment Analysis (DEA) and linear programming methods can be included in this category. Lengacher and Cammarata (2012) suggested a DEA model to evaluate and select Portfolio. As another model, Liu (2012) developed a weighted linear programming method for the site selection of distribution center problem.

Artificial Intelligence methods, as a third category, contain genetic algorithm, artificial neural network (ANN) and data mining methods. In this context, Guo et al. (2011) presented a Genetic Algorithm for Optimized Feature Selection with Resource Constraints in Software Product Lines. Similarly, Flintsch et al. (1998) suggested an artificial neural network model for project selection level pavement management system.

The last category is concerned with integrated approaches. There are so many studies about integrated methods for software selection problem in the literature. Onder and Dag (2013), proposed an approach based on AHP and improved TOPSIS for the supplier selection problem. In another study, Kuei-Yang Wu (2010) proposed an integrated approach of Fuzzy AHP for Constructing Evaluation Model for Sustainable Development in Community Health and Welfare. Eldrandaly and Naguib (2013) from another scale, proposed an integrated approach of expert system and AHP to select the best GIS software. Similarly, Zaidan et al. (2015) presented an approach based on integrated AHP and TOPSIS to select the optimal open-source EMR software packages.

In this study, we choose an integrated approach which combines AHP and TOPSIS methods. AHP method is an excellent MCDM technique as it provides a structure and hierarchy method for synthesizing software selection problems which is used to calculate the weight of selected criteria (Lin et al. 2007), and TOPSIS technique is employed to rank the alternative software based on their overall performance.

The proposed integrated methodology has many advantages compared to the previously proposed techniques in the literature as follows: (1) it is suitable for the evaluation and selection of ETL software to consider the decision-makers’ preferences depending on their knowledge and experiences; (2) the weights of multiple and conflicting criteria are obtained by using pair-wise comparisons according to preferences of the decision-makers; and (3) the global ranking of the software alternatives are provided.

## Multi-criteria decision-making methods

### Analytical hierarchy process method (AHP)

AHP is a multi-criteria decision making method that was proposed in the 1970s by Saaty. It has been used extensively for analyzing and structuring complex decision problems. The decision problem is first decomposed into different criteria (Dagdeviren et al. 2009). The AHP method can be used to assist decision-makers to calculate the weight for each criterion by using pair-wise comparison judgments (Liberatore and Nydick 1997; Yoo and Choi 2006; Panda et al. 2014).

AHP technique is a process that consists of the following steps (Saaty and Vargas 2001; Saaty 2008):

- Step 1: Structure the decision hierarchy taking into account the goal of the study and determine the criteria and sub-criteria.
- Step 2: Establish a set of all judgments in the comparison matrix in which the set of elements is compared to itself by using the fundamental scale of pair-wise comparison shown in Table 1.

**Table 1:** Scale of pair-wise comparison for AHP

Relative importance	Definition
1	Equal importance
3	Weak importance
5	Strong importance
7	Demonstrated importance over the other
9	Absolute importance
- Step 3: Determine the relative importance of factors by calculating the corresponding Eigenvectors to the maximum Eigen values of comparison.
- Step 4: Verify the consistency of judgments across the Consistency Index (CI) and the Consistency Ratio (CR).
  1 $$CI = \frac{\lambda_{\rm max} - n}{n - 1}$$
  where λ_max_ is the Eigen value corresponding to the matrix of pair-wise comparisons and n is the number of elements being compared.

Consistency ratio (CR) is defined by:

2 $$CR = \frac{CI}{RCI}$$

where, (RCI) is a random consistency index defined in Table 2.

**Table 2:** Average RCI values

Number of criteria (n)	RCI
1	0
2	0
3	0.58
4	0.90
5	1.12
6	1.24
7	1.32
8	1.41
9	1.45
10	1.49

A value of CR less than 0.1 is generally acceptable; otherwise the pair-wise comparisons should be revised to reduce incoherence.

### Technique for order preference by similarity to ideal solution (TOPSIS)

TOPSIS (Technique for Order Preference by Similarity to Ideal Solution) method was developed by Hwang, and Yoon (1981), for solving multiple criteria decision making (MCDM) problems based upon the concept that the chosen alternative should have the shortest distance to the positive ideal solution (A*) and the longest distance from the negative ideal solution (A−). For instance, the positive ideal solution maximizes the functionality and minimizes the cost, whereas the negative ideal solution maximizes the cost and minimizes the functionality. In the process of TOPSIS, the performance ratings and the weights of the criteria are given as exact values (Lengacher and Cammarata 2012). Recently, several interesting studies have focused on the TOPSIS technique and applied it in many fields, including supplier selection, tourism destination evaluation, financial performance evaluation, location selection, company evaluation, and ranking the carrier alternatives. Examples of these studies can be found in the literature such as ERP software selection (Huiqun and Guang 2012), customer-driven product design process (Lin et al. 2008), open-source EMR software packages (Zaidan et al. 2015). The steps of TOPSIS model are as follows (Tsaur 2011; Ding 2012):

- Step 1: Establish a decision matrix for the ranking.
- Step 2: Normalize the decision matrix using the following equation.
  3 $$e' = \left[ {\frac{{g_{j} (a_{i} )}}{{\sqrt {\sum\nolimits_{i = 1}^{m} {\left[ {g_{i} (a_{i} )} \right]^{2} } } }}} \right]; \, \quad {\text{i}} = 1, \, 2,\ldots,{\text{ m}};{\text{ and j}} = 1,2, \ldots, {\text{ n}}$$
  where *gi* is deterministic value of alternative *i* for criterion *j*.
- Step 3: Calculate the weighted normalized decision matrix by multiplying the normalized decision matrix with its associated weights as:
  4 $$e^{*}_{ij} = \pi_{j} \times e^{\prime}_{ij} ;\quad {\text{ i}} = 1,{ 2},\ldots,{ m};{\text{ and }}\,\, {\text{j}} = 1, 2,\ldots,{\text{ n}}$$
  where π_j_ is the weight of jth criterion.
- Step 4: Identify the positive ideal solution (A*) and negative ideal solution (A −).
  5 $$\begin{aligned} &{\text{Positive}}{:} \\ &A^{*} = \left\{ {e_{j}^{*} , j = 1, 2, \ldots, n} \right\} = \left\{ {e_{ 1}^{*} , e_{ 2}^{*} ,\ldots, e_{n}^{*} .} \right\};e_{j}^{*} = Max_{i} \left\{ {e_{ij}^{''} } \right\} \\ &A^{*} - = \{ Min_{i} .e_{ij}^{*} ,\,\, i = 1, \ldots, m; \,\, and\,\, j = 1,\ldots, n\} \\ \end{aligned}$$
  6 $$\begin{aligned} {\text{Negative}}{:} \hfill \\ A - =& \left\{Min_{i}\, {e_{ij}^{''} \left. {,\quad i = 1,\ldots,m;and \, j = 1,\ldots, n}\right\}} \right.;\quad e_{j^*} = Min_{i} \{ e_{ij}^{''} \} \hfill \\ A - =& \left\{ {e_{j^*} ,j = 1,2,\ldots,n} \right\} = \{ e_{1^*} ,e_{2^*}, \ldots, e_{n^*} \} \hfill \\ \end{aligned}$$
- Step 5: Determine the Euclidean distance of each alternative from the positive and negative ideal solutions.
  7 $$D_{i}^{*} = \sqrt {\sum\limits_{j = 1}^{n} \left(e^{*}_{ij} - e^{*}_{j} \right)^{2}} ,\quad {\text{ i}} = 1, 2, \ldots {\text{m}}$$
  8 $$D_{i}^{-} = \sqrt {\sum\limits_{j = 1}^{n} \left( e^{*}_{ij} - e_{j^{*}} \right)^{2}} ,\quad {\text{ i}} = 1, 2, \ldots {\text{m}}$$
- Step 6: Calculate the relative closeness coefficient of the *ith* alternative to ideal solution using the following equation:
  9 $$C_{i}^{*} = \frac{{D_{i}^{ - } }}{{D_{i}^{*} + D_{i}^{ - } }}, \, \quad {\text{i}} = 1, 2, \ldots,{\text{m}}$$
  With,  0 ≤ Ci* ≤ 1.
- Step 7: Rank all alternatives based on decreasing values of Ci* and selecting the optimal one.

### Proposed integrated multi-criteria decision methodology

Over the last decades, many researchers have devoted their effort to design the best methodologies for decision-making. The proposed methodology is designed in such a way that makes the use of MCDM techniques as efficient as possible. Two different techniques, namely AHP and TOPSIS, are combined in order to rank alternative software according to criteria. The reason for using the well-known AHP technique is to structure the decision hierarchy of the problem. Finally, to rank the alternatives, one of the most efficient MCDM techniques such as TOPSIS is used.

Hereafter, the main steps of the proposed integrated methodology to be elaborated by decisions-makers for the ETL software selection problem are as follows:

- Step 1: Define criteria and sub-criteria that are most affecting in the ETL software selection problem.
- Step 2: Construct a hierarchy decision model for the problem.
- Step 3: Determine the comparison matrix for each level (level of criteria and sub-criteria) by using AHP technique to obtain the local weight of each criterion and sub-criterion.
- Step 4: Determine the global weight by normalizing the local weight.
- Step 5: Use the TOPSIS technique to assess the alternatives where the most appropriate one can be easily selected.
- Step 6: Select the best ETL software alternative.

**Fig. 1:**
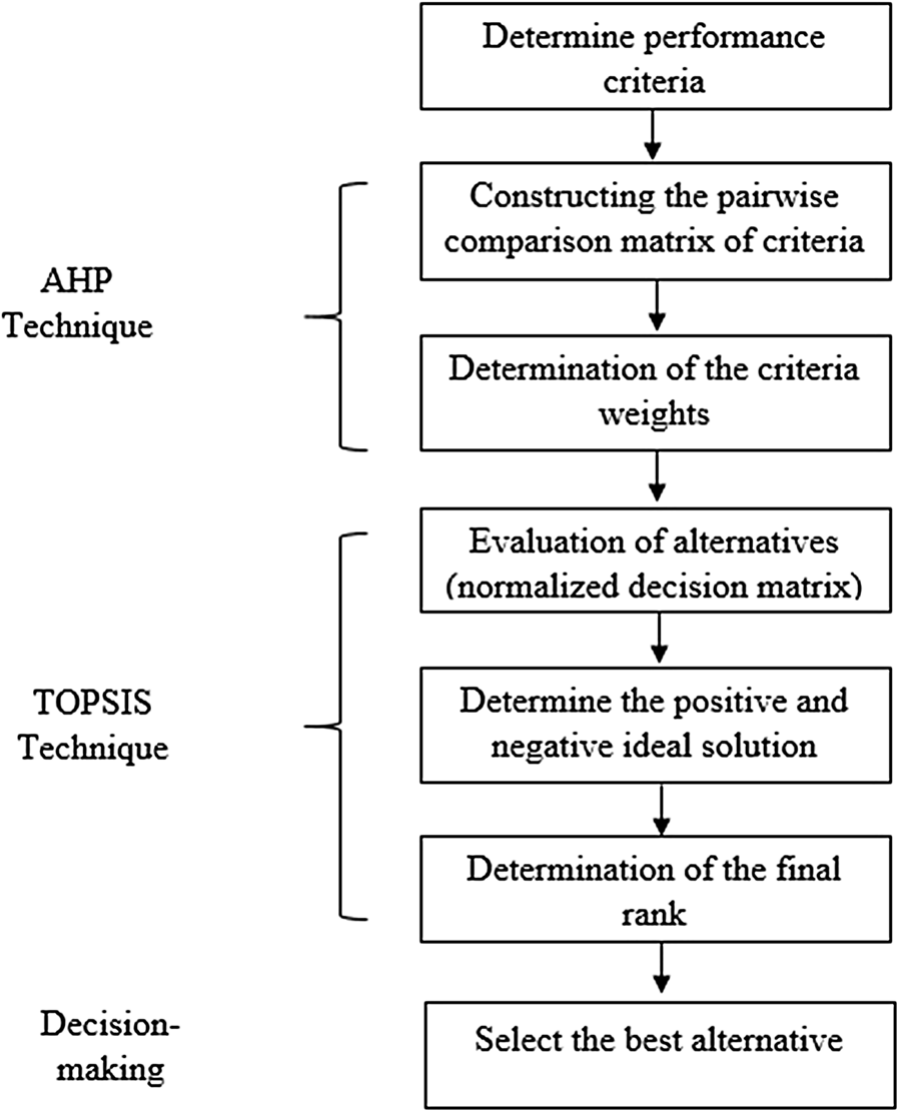
Proposed integrated methodology for ETL software selection problem

Figure 1 illustrates the process of the proposed integrated methodology to evaluate and select the ETL software.

### Numerical illustration

In this section, to better understand of the proposed integrated methodology, an application is provided. The ETL software selection decision is very important in long-term planning for any Business Intelligence project and is required due to various reasons, such as very expensive and open-source software available in the market. Then each tool uses its own methodology for extracting, transforming, and loading of data. Hence, decision-makers should select the software for a new project that not only performs well, but also is flexible enough to accommodate future changes in the project. The success or failure of any project depends on the consideration of various criteria when selecting new ETL software, such as: Functionality, Vendor, Usability, Cost, and Reliability (Wyatt et al. 2009; Kimball and Caserta 2004). In fact, conflicting criteria have high impact on the software performance. In this case, selection of the suitable ETL software involves the consideration of multiple feasible alternatives under multiple influential criteria. The problem is then solved by using the proposed approach explained in Fig. 1.

The integrated methodology is applied step by step to solve the ETL software selection problem for the BI project. After preliminary screening, five software: S1, S2, S3, S4 and S5, are chosen for further assessment. Hereafter, the main steps of the application of the proposed multi-criteria decision methodology for ETL software selection is elaborated.

Furthermore, the identification of the criteria and the sub-criteria is the first step of the proposed methodology. Based on the literature review and recent experiences of some specialists, in order to identify some general decision-making attributes (criteria) for selecting the best ETL software, we propose five main criteria and fifteen sub-criteria, which are the most prevalent and important in the selection of ETL software (Step 1).

The five main criteria Functionality, Vendor, Usability, Cost, and Reliability, are further broken down into sub-criteria. Functionality is characterized by compatibility, scheduler, category, support BI (Business Intelligence) and security. Vendor is divided into technical capability, reputation and provides permanent services. Additionally, Usability is associated with ease of use and completeness of the GUI (graphical user interface), while Cost criterion is broken down into maintenance cost, consultant expense and price. Reliability is finally divided into stability and recovery ability.

**Fig. 2:**
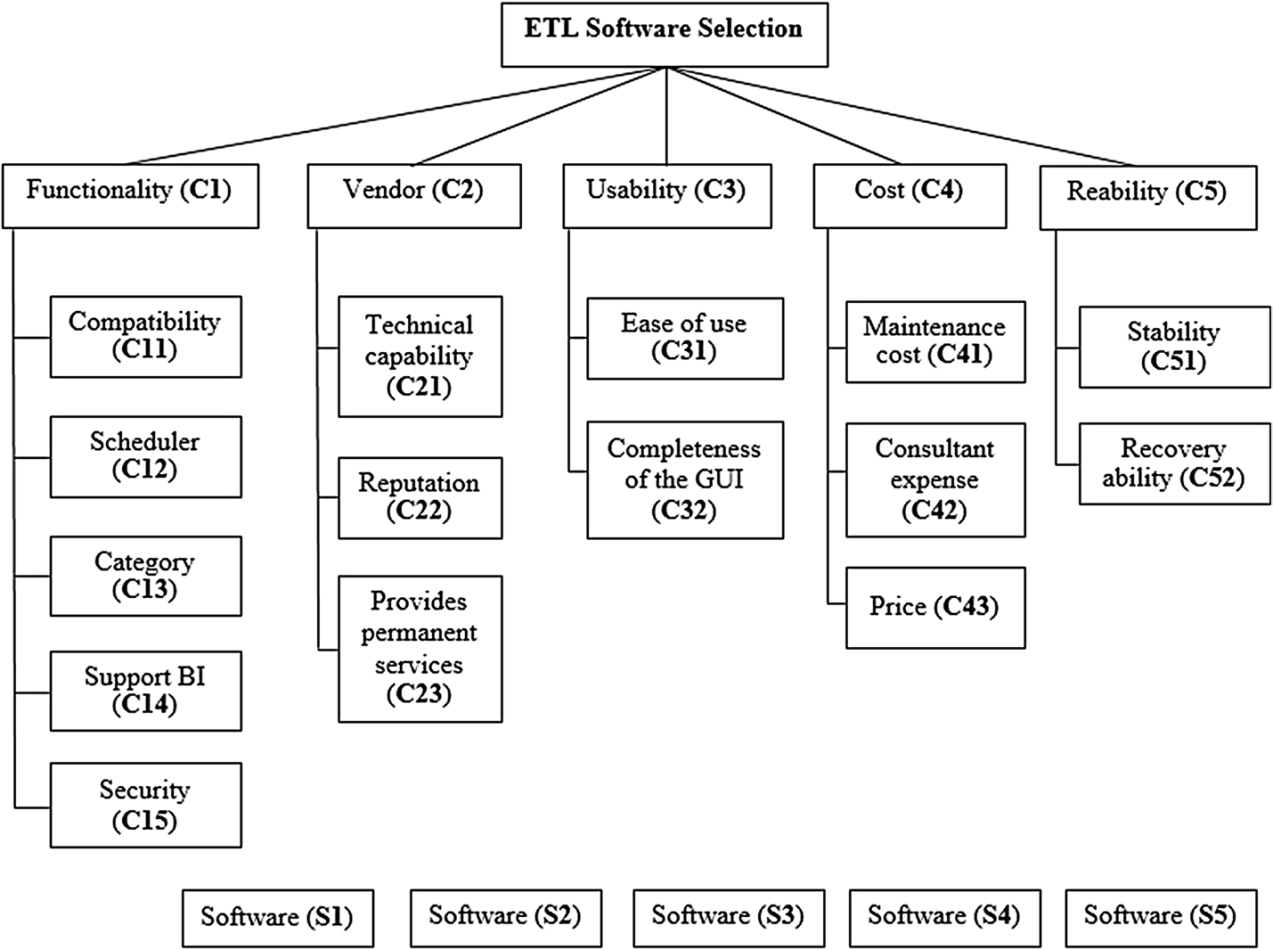
Hierarchy model of ETL software selection

In the following, decision-makers follow the computational procedure of weights for selected criteria using AHP method, and then rank the alternatives with TOPSIS method. In the first step of AHP technique, we developed a hierarchy model of ETL software selection based on the criteria, sub-criteria, and alternatives (Step 2). As shown in Fig. 2, the highest and the lowest levels of the hierarchy denote the overall objective (selecting the most appropriate ETL software) and the software proposed (S1, S2, S3, S4, and S5) respectively. The five main criteria are included in the second level (Mousavi et al. 2012) and are further broken down into sub-criteria in the third level.

Once the hierarchy has been constructed through the AHP technique, the next step is dedicated to obtain the weights of elements at each level of the hierarchy. A set of comparison matrices of all elements for each level of the hierarchy with respect to elements of the higher level are realized. The preferences of decision-makers are identified using Saaty scale (Saaty 2008) as shown in Table 1.

The initial pair-wise comparison matrix for the main criteria provided by decision makers is presented in Table 3. Moreover, the matrix of sub-criteria of the first main criterion C1 (i.e., Functionality) presented by decision-makers is given in Table 4 (Step 3). Other comparison matrices of the sub-criteria are not shown here. But, Table 5 shows all weight vectors calculated by pair-wise comparisons as similar to C1 in Table 4 (Step 4). Ultimately, the consistency ratio (CR) of each pair-wise comparison judgment matrices is also shown below in each matrix. It can be seen that the CR of each matrix is less than 0.1.

**Table 3:** The comparison matrix of criteria

Criteria no	C1	C2	C3	C4	C5	Weights
C1	1	3	3	5	3	0.38
C2	1/3	1	1/5	1	1/3	0.07
C3	1/3	5	1	3	3	0.31
C4	1/5	1	1/3	1	1	0.09
C5	1/3	3	1/3	1	1	0.14
					CR:	0.08166

**Table 4:** The comparison matrix of sub-criteria with respect to criteria C1

Criteria (C1)	C11	C12	C13	C14	C15	Weights
C11	1	3	1	1/5	1/3	0.13
C12	1/3	1	1/3	1/5	1/3	0.05
C13	1	3	1	1/5	1/3	0.13
C14	5	5	5	1	3	0.45
C15	3	3	3	1/3	1	0.24
					CR:	0.06065

**Table 5:** The normalized sub-criteria weightings

Criteria	Level one	Sub-criteria	Level two
Functionality	0.38	Compatibility	0.05
		Scheduler	0.02
		Category	0.05
		Support BI	0.17
		Security	0.09
Vendor	0.07	Technical capability	0.026
		Reputation	0.01
		Provides permanent service	0.034
Usability	0.31	Ease of use	0.254
		Completeness of the GUI	0.056
Cost	0.09	Cost of maintenance	0.033
		Consultant expense	0.013
		Price	0.044
Reliability	0.14	Stability	0.035
		Recovery ability	0.105

At the final step, TOPSIS method is applied in order to rank the alternative software. The global weights of each sub-criterion which are calculated by AHP can be used as the input in TOPSIS method. Then using the scale in Table 1, the decision-makers are asked to evaluate the alternatives according to each sub-criterion (Table 6) (Step 5.1). The second step in TOPSIS technique is the normalizing of the aggregate ratings matrix, using the Eqs. (3), (4), as illustrated in Table 7 and thereafter, by using the Eqs. (5), (6), we can calculate the positive and negative ideal solutions (i.e., A* and A−) for the five alternatives (Step 5.2).

**Table 6:** Input values of the TOPSIS analysis

Criteria	Weight	S1	S2	S3	S4	S5
C11	0.0494	5	7	9	3	5
C12	0.0190	5	5	3	9	7
C13	0.0494	3	3	5	7	3
C14	0.1710	3	5	9	3	7
C15	0.0912	3	5	9	3	7
C21	0.0259	5	7	5	3	3
C22	0.0098	5	7	3	5	5
C23	0.0343	7	3	5	3	7
C31	0.2542	3	5	7	7	3
C32	0.0558	5	9	9	9	5
C41	0.0333	9	9	3	7	5
C42	0.0126	7	7	3	5	9
C43	0.0441	3	5	3	5	7
C51	0.0350	5	3	5	3	3
C52	0.1050	7	5	7	7	5

**Table 7:** The weighted normalized decision matrix

Criteria	S1	S2	S3	S4	S5		A*	A−
C11	0.018	0.025	0.032	0.010	0.018	+	0.032	0.010
C12	0.007	0.007	0.004	0.012	0.01	+	0.012	0.004
C13	0.015	0.015	0.024	0.034	0.015	+	0.034	0.015
C14	0.04	0.065	0.117	0.04	0.091	+	0.117	0.04
C15	0.020	0.034	0.062	0.020	0.048	+	0.062	0.020
C21	0.012	0.017	0.012	0.007	0.007	+	0.017	0.007
C22	0.004	0.006	0.002	0.004	0.004	+	0.006	0.002
C23	0.020	0.008	0.014	0.008	0.020	+	0.020	0.008
C31	0.064	0.107	0.149	0.149	0.064	+	0.149	0.064
C32	0.016	0.029	0.029	0.029	0.016	+	0.029	0.016
C41	0.019	0.019	0.006	0.015	0.010	−	0.006	0.019
C42	0.006	0.006	0.002	0.004	0.007	−	0.002	0.007
C43	0.012	0.020	0.012	0.020	0.028	−	0.012	0.028
C51	0.02	0.012	0.02	0.012	0.012	+	0.019	0.012
C52	0.052	0.037	0.052	0.052	0.037	+	0.052	0.037

The ranking of alternative software are calculated by using the Eqs. (7), (8), and (9). Table 8 shows the evaluation results and final ranking of alternatives. Therefore, the best alternative is the one with the shortest distance to the positive ideal solution and with the longest distance to the negative ideal solution. The proposed model results show that software 3 is the best alternative with Ci value of 0.0084 (Step 6).

**Table 8:** The final evaluation and ranking of alternatives

	D*	D^−^	Ci	Rank
S1	0.128	0.0000	0.0000016	5
S2	0.077	0.000013	0.00017	4
S3	0.030	0.000256	0.0084	1
S4	0.094	0.000066	0.0007	2
S5	0.096	0.000018	0.00019	3

**Fig. 3:**
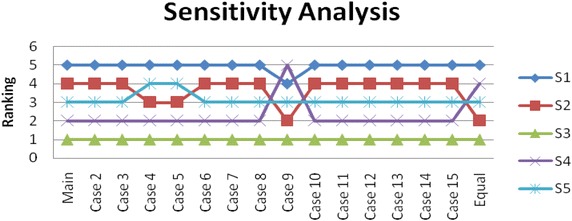
Sensitivity analyses under different criteria weights

A sensitivity analysis is performed to analyze the two phases AHP and TOPSIS methodology proposed in this paper. For this reason, the criteria weights obtained from AHP are exchanged between two criteria while the others are constant. In other words, the weight of the first criterion C11 is exchanged with C12, C13 … and C52, sequentially, while the others are constant. For each case, the A*, A− and the closeness coefficient (Ci) are calculated to display the new results and hence sixteen combinations (by adding the equal weight criterion) of the fifteen sub-criteria are analyzed (Gumus 2009). Then, the details of all the cases are summarized in Table 9 and the closeness coefficient with ranking of the alternatives are shown both in Table 10 and graphically represented in Fig. 3.

**Table 9:** Details for sensitivity analysis

Cases	C11	C12	C13	C14	C15	C21	C22	C23	C31	C32	C41	C42	C43	C51	C52
1 (main)	0.05	0.02	0.05	0.17	0.09	0.02	0.01	0.03	0.25	0.05	0.03	0.01	0.04	0.03	0.10
2	0.02	0.05	0.05	0.17	0.09	0.02	0.01	0.03	0.25	0.05	0.03	0.01	0.04	0.03	0.10
3	0.05	0.02	0.05	0.17	0.09	0.02	0.01	0.03	0.25	0.05	0.03	0.01	0.04	0.03	0.10
4	0.17	0.02	0.05	0.05	0.09	0.02	0.01	0.03	0.25	0.05	0.03	0.01	0.04	0.03	0.10
5	0.09	0.02	0.05	0.17	0.05	0.02	0.01	0.03	0.25	0.05	0.03	0.01	0.04	0.03	0.10
6	0.02	0.02	0.05	0.17	0.09	0.05	0.01	0.03	0.25	0.05	0.03	0.01	0.04	0.03	0.10
7	0.01	0.02	0.05	0.17	0.09	0.02	0.05	0.03	0.25	0.05	0.03	0.01	0.04	0.03	0.10
8	0.03	0.02	0.05	0.17	0.09	0.02	0.01	0.05	0.25	0.05	0.03	0.01	0.04	0.03	0.10
9	0.25	0.02	0.05	0.17	0.09	0.02	0.01	0.03	0.05	0.05	0.03	0.01	0.04	0.03	0.10
10	0.05	0.02	0.05	0.17	0.09	0.02	0.01	0.03	0.25	0.05	0.03	0.01	0.04	0.03	0.10
11	0.03	0.02	0.05	0.17	0.09	0.02	0.01	0.03	0.25	0.05	0.05	0.01	0.04	0.03	0.10
12	0.01	0.02	0.05	0.17	0.09	0.02	0.01	0.03	0.25	0.05	0.03	0.05	0.04	0.03	0.10
13	0.04	0.02	0.05	0.17	0.09	0.02	0.01	0.03	0.25	0.05	0.03	0.01	0.05	0.03	0.10
14	0.03	0.02	0.05	0.17	0.09	0.02	0.01	0.03	0.25	0.05	0.03	0.01	0.04	0.05	0.10
15	0.10	0.02	0.05	0.17	0.09	0.02	0.01	0.03	0.25	0.05	0.03	0.01	0.04	0.03	0.05
Equal	0.06	0.06	0.06	0.06	0.06	0.06	0.06	0.06	0.06	0.06	0.06	0.06	0.06	0.06	0.06

**Table 10:** Results of sensitivity analysis

Conditions	Alternative software					Ranking
	S1	S2	S3	S4	S5	
Case 1 (main)	0.0000016	0.00017	0.0084	0.0007	0.00019	S3-S4-S5-S2-S1
Case 2	0.0000016	0.00016	0.0067	0.00079	0.00020	S3-S4-S5-S2-S1
Case 3	0.0000016	0.00017	0.0084	0.0007	0.00019	S3-S4-S5-S2-S1
Case 4	0.0000091	0.00044	0.0080	0.00072	0.00005	S3-S4-S2-S5-S1
Case 5	0.0000027	0.00022	0.0083	0.0007	0.00015	S3-S4-S2-S5-S1
Case 6	0.0000018	0.00018	0.0080	0.0007	0.00018	S3-S4-S5-S2-S1
Case 7	0.0000018	0.00018	0.0070	0.0007	0.00019	S3-S4-S5-S2-S1
Case 8	0.0000026	0.00016	0.0080	0.0007	0.00020	S3-S4-S5-S2-S1
Case 9	0.0000260	0.00070	0.0141	0.000007	0.00036	S3-S2-S5-S1-S4
Case10	0.0000017	0.00017	0.0085	0.0007	0.00019	S3-S4-S5-S2-S1
Case11	0.0000031	0.00018	0.0074	0.0007	0.00019	S3-S4-S5-S2-S1
Case12	0.0000026	0.00017	0.0067	0.0007	0.00022	S3-S4-S5-S2-S1
Case13	0.0000016	0.00017	0.0080	0.0007	0.00020	S3-S4-S5-S2-S1
Case14	0.0000019	0.000162	0.0082	0.0007	0.00018	S3-S4-S5-S2-S1
Case15	0.0000031	0.00022	0.0113	0.00064	0.00018	S3-S4-S2-S5-S1
Equal	0.00007	0.00021	0.00028	0.00013	0.00021	S3-S2-S5-S4-S1

From Table 10 and Fig. 3, it can be seen that the first case describes the original results of the integrated methodology. Also, out of sixteen cases, alternative software S3 has the highest score in all cases. Moreover, the results of the sensitivity analysis indicate that the alternatives’ ranking has changed significantly according to equal weights of the criteria. Therefore, we can say that, based on the evaluations obtained, our decision making process is relatively insensitive to the criteria weights with S3 emerging as the winner of all the cases (Mousavi et al. 2012).

### Proposed software

As can be easily proved, AHP and TOPSIS require many time-consuming calculations, depending upon the number of the criteria, sub-criteria and alternatives that are necessary to make all calculations in order to reach the final solution (Duran 2011). As the number of criteria increases, the dimension of the problem expands. This could lead to a great number of mathematical operations. Therefore, software aid may be very useful to automatically carry out the methodology process. A software prototype for this methodology (AHP-TOPSIS) application is developed. This software is programmed by using JAVA on a PC platform. The operation sequence will be demonstrated in the following paragraphs through the use of several screenshots.

**Fig. 4:**
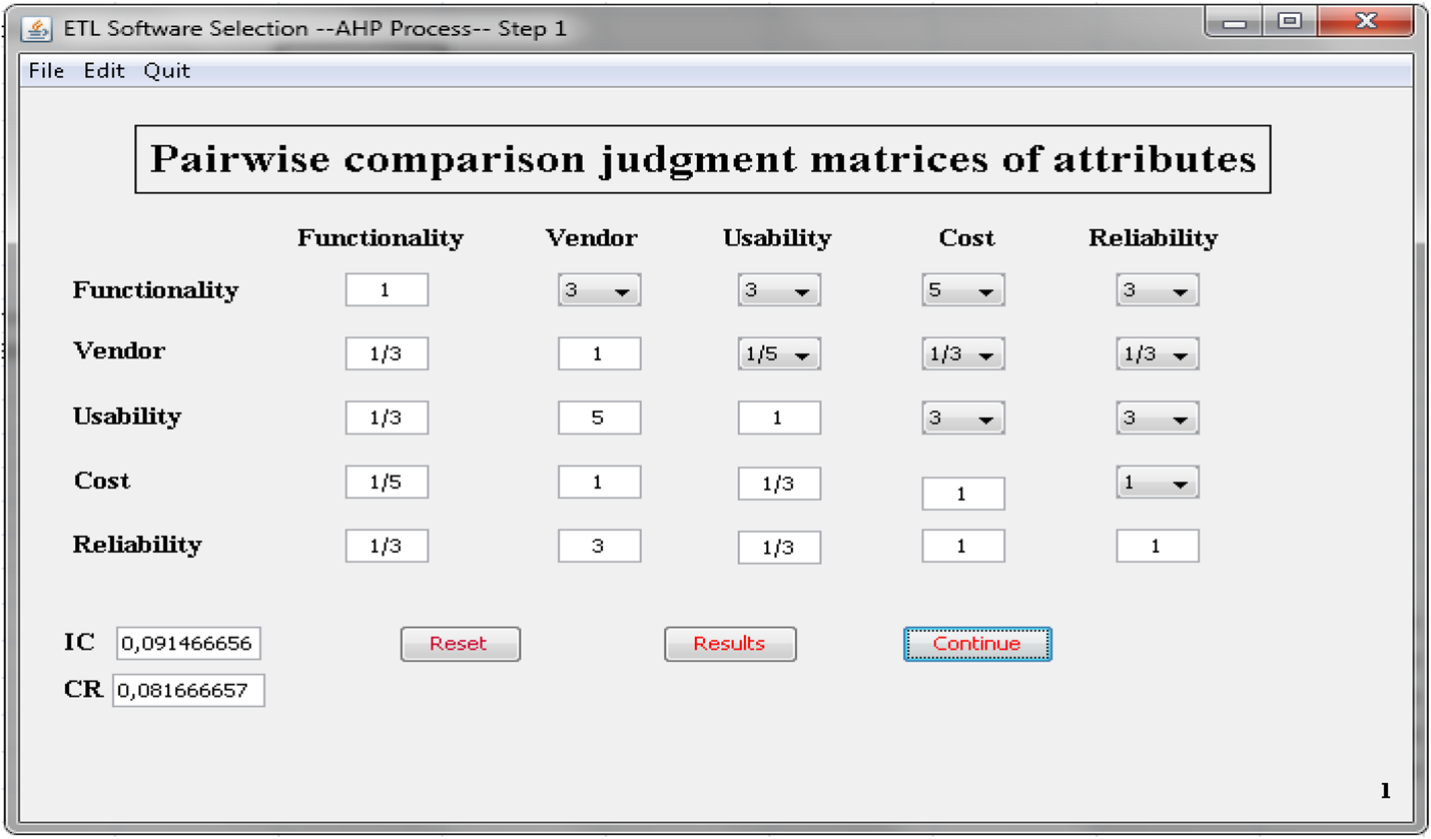
Screenshot of comparison matrix

**Fig. 5:**
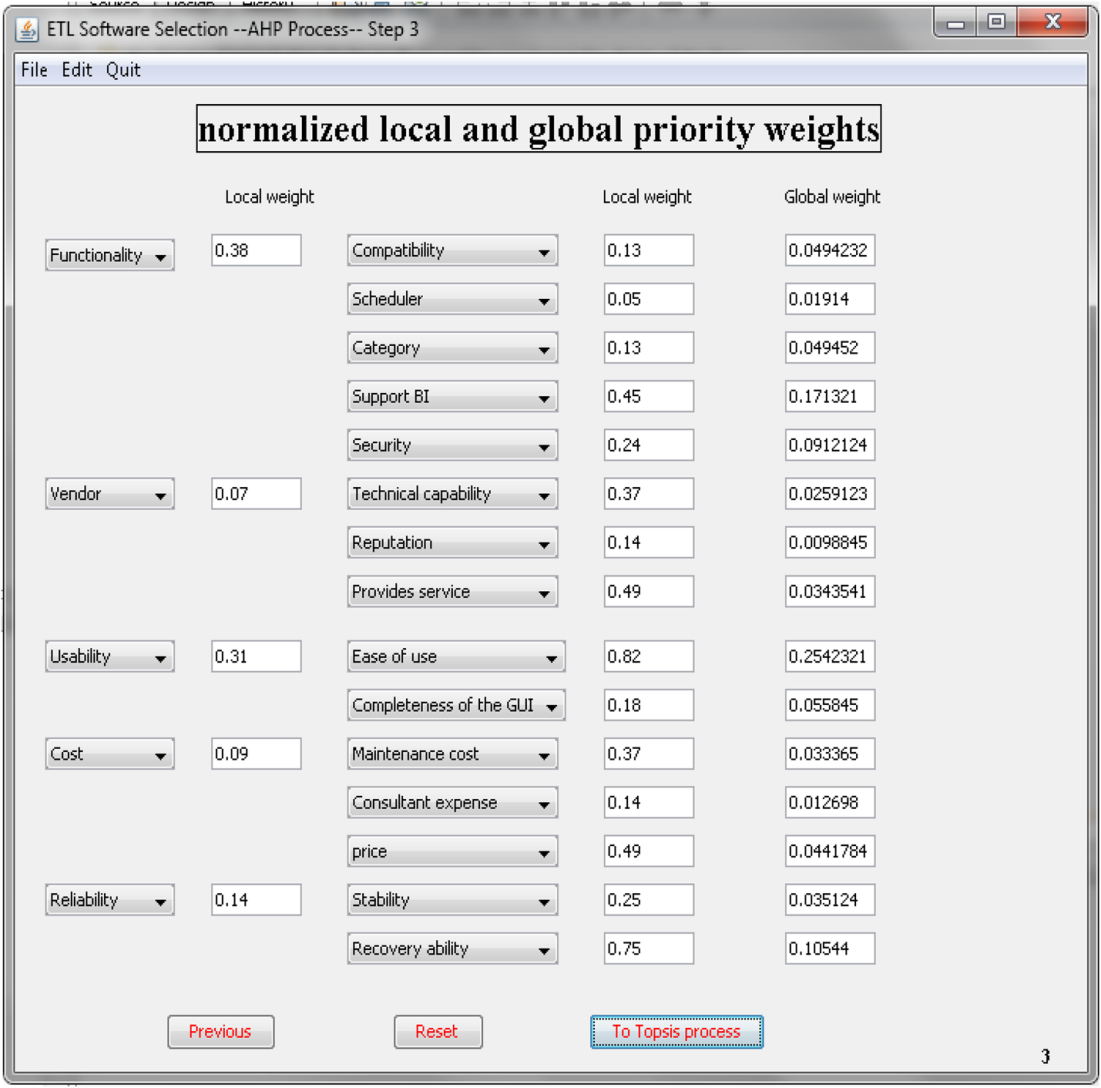
First phase analysis results

Initially, the user must supply the criteria and sub-criteria chosen for the evaluation of the ETL software. The software prototype keeps a series of attributes that the user can select to perform the comparison analysis in a database. Moreover, the database contains a set of generic criteria and sub-criteria labeled as ‘‘criterion i’’ and “sub-criterion j” where ‘‘i and j’’ stands for the number of a given criterion and sub-criterion in order. Then, the user must fill the pair-wise comparisons matrix for the criteria and sub-criteria. Figure 4 shows the Combo Box where the analyst can input the pair-wise comparisons among the software’s criteria (using the scale of AHP). Once the comparison matrix is entirely filled with importance values, the ratio of consistency (CR) is computed automatically. In Fig. 5, the system provides the Eigenvector of the five criteria and the fifteen sub-criteria according to the information inputted by the user.

**Fig. 6:**
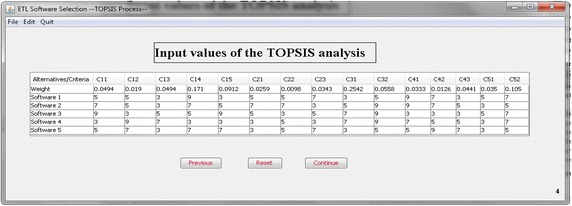
Input values of the TOPSIS analysis

**Fig. 7:**
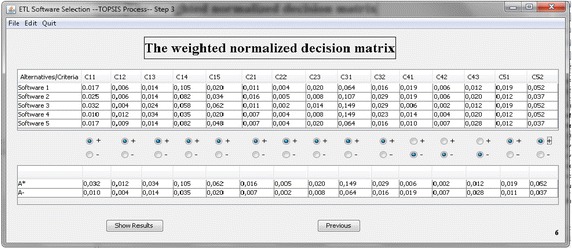
Weighted evaluations of the alternative software and calculation of positive and negative ideal solutions

In the second part of the software, concerning the application of TOPSIS method, it is assumed that users must input the pair-wise comparisons of the five specific ETL software proposed. This task is made accordingly to each one of the considered sub-criteria (Fig. 6). In the next step, the weighted normalized decision matrix is calculated, and the system must identify the positive A* and negative A− ideal solutions (Fig. 7).

**Fig. 8:**
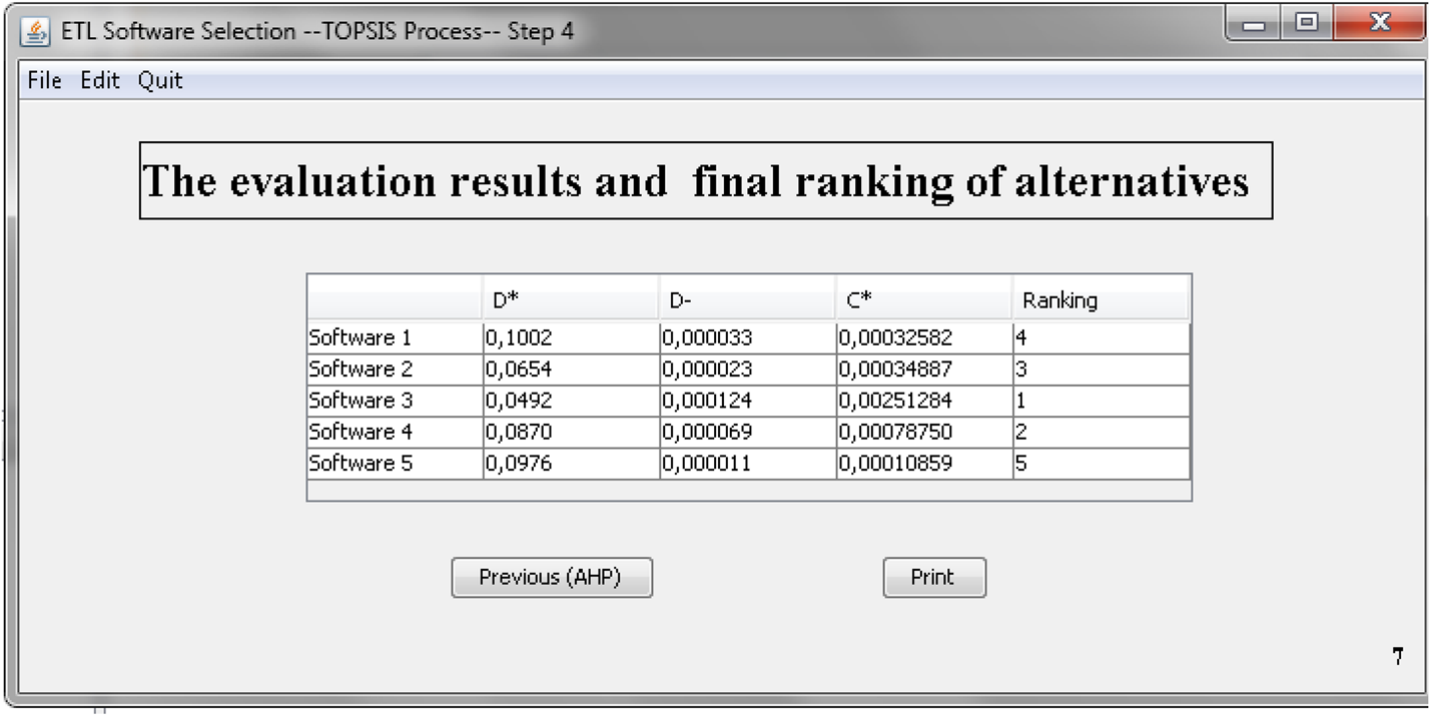
Final results of TOPSIS

Finally, the software finds the scores of the ETL alternatives with respect to criteria and sub-criteria. It displays the results using the calculation of the distance between positive and negative ideal solutions. Therefore, the values and the priority scores for the ETL software alternatives are obtained. The ranked list indicates that in this case the alternative software with the higher value of Ci has to be selected by the users as illustrated in Fig. 8.

## Conclusion

Nowadays, ETL software play an essential role in Business Intelligence (BI) projects, selecting suitable ETL software has become one of the most important issues for starting a BI project. Our contribution presents an application of methodology based on a hybrid multi-criteria decision making process. The methodology consists of two techniques: analytical hierarchy process (AHP) and technique for order preference by similarity to ideal solution (TOPSIS). This methodology is tested by an example and it was found that it functions satisfactorily. Five software of ETL are chosen to demonstrate how the approach is applied and lead to the selection of the software consistent with the maximization of the underlying techniques for all the decision-makers. Additionally, an AHP-TOPSIS methodology based software for selecting ETL software was proposed.

Although, the integrated methodology and the software prototype are introduced for ETL software selection problem, it can also be applied for any other software selection problem involving multiple and conflicting criteria. For further studies, different techniques of MCDM, such as ELECTRE, MACBETH, PROMETHEE, can be used and comparison of the results can be explored. Probably, the pair-wise comparison of the proposed methodology seems insufficient and too imprecise to capture exactly the judgments of decision-makers, which is serious limitation. Regarding this point, the methodology can be considered under the fuzzy environment.
